# Chain Formation and Phase Separation in Ferrofluids: The Influence on Viscous Properties

**DOI:** 10.3390/ma13183956

**Published:** 2020-09-07

**Authors:** Alexey O. Ivanov, Andrey Zubarev

**Affiliations:** 1 Department of Theoretical and Mathematical Physics, Ural Federal University, Lenin Ave. 51, 620000 Ekaterinburg, Russia; Alexey.Ivanov@urfu.ru; 2 M.N. Mikheev Institute of Metal Physics of the Ural Branch of the Russian Academy of Sciences, 620990 Ekaterinburg, Russia

**Keywords:** ferrofluid, chain aggregates, phase separation, rheological properties

## Abstract

Ferrofluids have attracted considerable interest from researchers and engineers due to their rich set of unique physical properties that are valuable for many industrial and biomedical applications. Many phenomena and features of ferrofluids’ behavior are determined by internal structural transformations in the ensembles of particles, which occur due to the magnetic interaction between the particles. An applied magnetic field induces formations, such as linear chains and bulk columns, that become elongated along the field. In turn, these structures dramatically change the rheological and other physical properties of these fluids. A deep and clear understanding of the main features and laws of the transformations is necessary for the understanding and explanation of the macroscopic properties and behavior of ferrofluids. In this paper, we present an overview of experimental and theoretical works on the internal transformations in these systems, as well as on the effect of the internal structures on the rheological effects in the fluids.

## 1. Introduction

Ferrofluids (magnetic fluids) are nanodispersed suspensions of single-domain ferro- or ferrimagnetic particles in a carrier liquid. To prevent irreversible colloidal aggregation of the particles, they are covered with surface layers that screen this interaction. Usually, the particles are prepared using iron oxides (magnetite or maghemite), though sometimes cobalt is used. As a rule, the carrier liquid is either an organic (kerosene, technical oil) or non-organic polar liquid (usually water). Ferrofluids have attracted considerable interest from researchers and engineers due to their rich set of unique physical properties that show promise for many industrial and biomedical applications.

All known ferrofluids are polydisperse. Typically, the mean diameter of the particles is about 10 nm; however, the distribution over their sizes can be quite broad and include particles with diameters of 20 nm or more. Note that the typical thickness of a screening surface layer is 2–2.5 nm. Estimates show that the magnetic interaction between the colloidal stabilized iron oxide particles with a diameter of about 10 nm is weak; at room temperature, this interaction can be ignored.

However, the energy of interaction between the relatively large particles, whose diameter is more than 15–17 nm, can significantly exceed the thermal energy *kT*, where *k* is the Boltzmann constant and *T* is the temperature. If the concentration of the particles is large enough, they can experience structural transformations and form various heterogeneous structures (linear chains, closed rings, branched forks and nets, etc.). The particles can also experience concentration phase transitions of the “gas–liquid” or “gas–solid” types. A magnetic field, macroscopic flow, and other external factors can induce and strongly affect the structural transformations in ferrofluids. Note that the concentration threshold for the structuring of the particles depends on their magnetic moments, the thickness of the surface layers on the particles, the applied magnetic field, the temperature, and other parameters of the system. This is why it is impossible to determine some universal threshold concentration. Usually, it varies from a fraction of a percent to several or even 10%.

The macroscopic physical properties of ferrofluids are determined by the internal structures and transition scenarios of the various morphologies of the particles’ spatial dispositions. It enables controlling the properties and features of these systems’ behaviors over a wide range of magnitudes by using a magnetic field and other external influences. In turn, it allows for ferrofluids to be applied to promising smart materials for many high-tech industrial and biomedical applications: magnetofluidic bearings and dampers; positioning systems; sensors, including biosensors; cooling systems; wormlike locomotives; actuators; magnetic nanoparticles that are injected in an organism with a ferrofluid to be used as labels for monitoring of biological binding reactions; for drug delivery and targeting; in magnetic hyperthermia therapy of oncological decease.

In this paper, we review experimental, theoretical, and computational studies on the structural transformations in ferrofluids, as well as their effects on the magnetic and dynamic properties of these systems. Discussions of the methods of ferrofluid synthesis, a basic introduction into the physics of these systems, and their practical applications can be found in books [1,2,3,4,5,6] and reviews [7,8,9,10,11,12,13,14,15].

## 2. Main Structures and Approaches

The energy of the magnetic interaction between two uniformly magnetized spherical particles with permanent magnetic moments ***m*_1_** and ***m*_2_** is equal to the energy $U_{dd}$ of the interaction between two point dipoles situated at the centers of the particles:

(1) $$U_{dd}\left(r,m_{1},m_{2}\right)=-\frac{\mu_{0}}{4\pi }\frac{3\left(m_{1}\cdot r\right)\left(m_{2}\cdot r\right)-r^{2}\left(m_{1}\cdot m_{2}\right)}{r^{5}},$$

where $\mu_{0}$ is the vacuum magnetic permeability and ***r*** stands for the radius vector that links the centers of the particles. It is convenient to introduce a dimensionless parameter *λ* for the interaction energy between two identical closely situated particles, each with magnetic moment *m* and diameter *d*:

(2) $$\lambda =\frac{\mu_{0}}{4\pi }\frac{m^{2}}{d^{3}kT}$$

If the central colloidal attraction of the particles is well screened, their aggregation can take place only if λ is significantly more than one. Otherwise, the thermal effects destroy any aggregates and clusters.

Because the size of the particles in ferrofluids is much smaller than the wavelength of visible light, the separate particles and branched structures consisting of the chain segments cannot be detected using optical observations. However, they have been detected using electronic microscopy (see, for example, [16,17,18]) and have been visualized in many computer simulations (for example, [18,19,20,21,22,23,24,25,26,27,28,29] and an overview in [5]).

Possibly the first theoretical study of ferrofluid chaining is presented in [30]. A system of identical spheres with dipole–dipole interactions was considered using the approximation of pair correlations. The anisotropic correlations between the particles’ positions were interpreted as chain-like structures, where the mean length of the correlation was taken to be the mean size of the chain. The following estimates for the mean number of particles in the chains were obtained

(3) $$<n>=\frac{3\lambda^{3}}{3\lambda^{3}-2\varphi \exp\left(2\lambda \right)}\;\mathrm{for}\;H=0,$$

(4) $$<n>=\frac{3\lambda^{2}}{3\lambda^{2}-2\varphi \exp\left(2\lambda \right)}\;\mathrm{for}\;\;H\to \infty ,$$

where *H* is the magnetic field inside the sample and φ is the volume concentration of the particles. It was noted in [30] that, because of long-range decay of the potential $U_{dd}\left(r,m_{1},m_{2}\right)$, the integral $\int \left[1-exp\left(-\frac{U_{dd}}{kT}\right)\right]dr$, which takes place in statistical calculations of the thermodynamic characteristics of ferrofluids, converges conditionally; its magnitude, and even sign, depends on the coordinate system used for the evaluation of this integral and on the order of integration over components of the vector ***r***. This conclusion is very important for the development of theoretical approaches used to study the physics of ferrofluids. Analysis has shown that the theory presented in [30] considers the homogeneous fluctuations (“clouds”) of a local density of the particles in ferrofluids. Therefore, the validity of its application for the description of heterogeneous chains and other aggregates is, at best, disputable.

Another approach used for the description of heterogeneous chains was suggested in [31,32,33]. The chains were considered to be flexible polymer-like aggregates with some distribution function over the number of particles in the aggregate. Unfortunately, the results of [31,32,33] are presented in mathematically complicated forms, which are difficult for practical use. The authors provide a qualitative discussion of the results that take into account, e.g., the experimental observations reported in [34,35,36,37], but a comparison of these models using data from either laboratory experiments or computer simulations is lacking.

**Figure 1:**
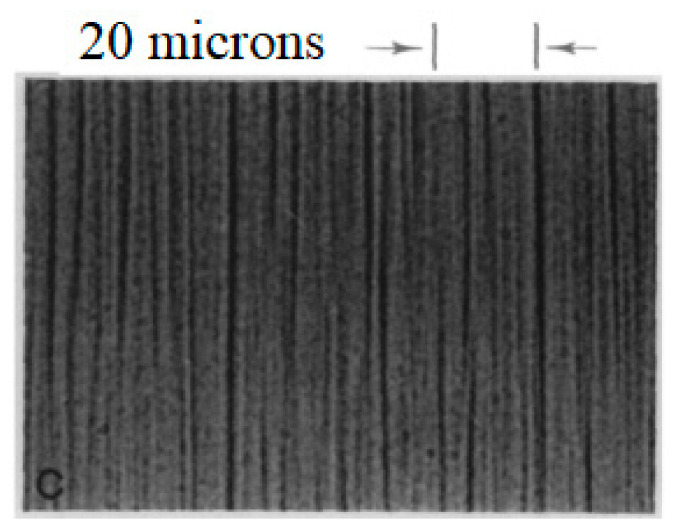
Typical photo [34] of the drop-like aggregates aligned along an applied magnetic field.

Dense drop-like aggregates of magnetic nanoparticles were observed in many works, for example, in [34,38,39,40,41,42,43,44,45,46,47,48,49,50,51,52,53,54,55]. The typical size of the drops (several microns or more) allows for observing them using an optical microscope. An image of these drops is given in Figure 1.

In the absence of an external magnetic field, the drops are roughly spherical; due to the action of a magnetic field, they become aligned along the field. The shape of the drop is determined by the balance between the “capillary” forces on the drop’s surface, which tend to transform the drop into a sphere, and the forces of the demagnetizing field, which tend to elongate the drop and transform it into a very narrow needle. In the simplest approximations, these drops can be modeled as ellipsoids of revolution. Regarding this approximation, detailed calculations of the drop shape can be found in [40,41,56]. The ellipsoidal approximation is in good agreement with experimental observations [40,41] in weak-to-moderate magnetic fields. Note that the capillary forces are proportional to the area of the drop surface, while the demagnetizing forces are proportional to the drop volume. This is why the drops with a relatively large volume are more elongated than the smaller ones.

**Figure 2:**
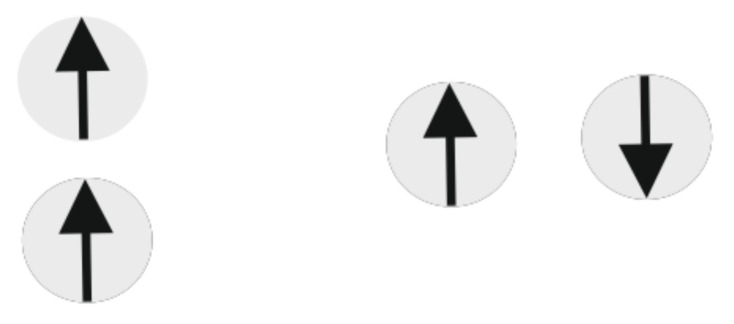
Illustration of the “head-to-tail” (left) and “side-by-side” (right) relative alignments of the particles.

The first theoretical descriptions of the bulk condensation in ferrofluids treated this phenomenon as a gas–liquid phase transition in the system of the magnetically interacting particles [57,58] (see also [2]). In these models, the magnetic interaction between the particles is taken into consideration by using the classical self-consisting Weiss model that was developed for para- and ferromagnetic systems with the exchange interaction between the atoms of the material. This is why the models [36,37] predict the second-order paramagnetic–ferromagnetic phase transition, which, to the best of our knowledge, has never been observed in real ferrofluids. The principal difference between the ferromagnetic exchange interaction and the dipole–dipole interaction is that the first one creates a parallel orientation of the magnetic moments of the interacting particles (atoms), independently of their relative spatial positions, whereas the dipole–dipole interaction induces a co-aligned orientation if the particle moments are arranged “head to tail” and an antiparallel orientation if they are arranged “side by side” (see Figure 2). That is why the application of self-consisting models, which have been successfully used for systems with exchange interactions, requires careful attention regarding the dipole–dipole interparticle interaction in ferrofluids.

**Figure 3:**
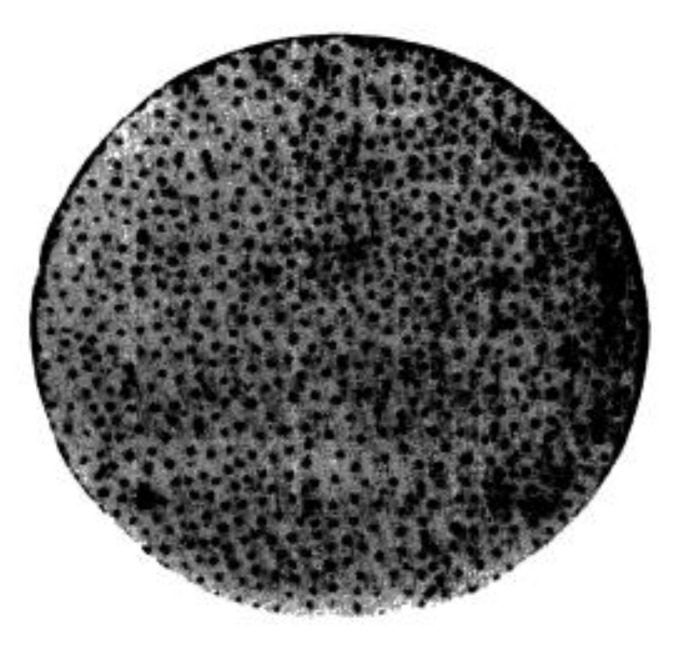
Photo [59] of the dense cylindrical domains in a gap that is filled with ferrofluid. The field is perpendicular to the figure plane. Published with permission of the American Physical Society (license RNP/20/AUG/029405).

An interesting peculiarity of the condensation phase transitions in ferrofluids, namely, filling thin gaps, has been detected in [39,45,49,59]. As is well known, at the phase separation, the nuclei of a new phase tend to coalesce into one massive bulk. The physical reason for this effect is the tendency to reduce the interface area, and therefore, create a corresponding reduction in the system’s energy. However, the phase separation of ferrofluids in thin gaps under the action of a magnetic field perpendicular to the gap plane leads to the appearance of stable discrete cylindrical domains of the dense phase. Some photos of these domains are shown in Figure 3.

The physical explanation of this phenomenon involves the balance between the surface tension effects, which tend to coalescence these domains, and the effects of their demagnetizing field, which tend to transform the domains into very thin “needles” [60]. As a result, the discrete thermodynamically equilibrium domains, whose diameter depends on the applied magnetic field strength and the gap thickness, take place in the system.

## 3. Chain-Like Structures and Their Effect on Macroscopic Properties of Ferrofluids

The first theoretical models of ferrofluids (for an overview, see [1,61]) considered the approximation of single, non-interacting particles. In this approximation, the magnetization *M* of a suspension of identical spherical particles is determined using the Langevin function *L*(*x*):

(5) $$L\left(x\right)=\coth\left(x\right)-x^{-1}\;,\;M=m\frac{\varphi }{v_{p}}L\left(\kappa \right),\;\kappa =\mu_{0}\frac{mH}{kT},$$

where $v_{p}$ is the particle volume and *κ* is the dimensionless magnetic field. The effective viscosity of the magnetic colloid can be estimated using:

(6) $$\eta =\eta_{0}\left(1+\frac{5}{2}\varphi +\frac{3}{2}\varphi \frac{\kappa L^{2}\left(\kappa \right)}{\kappa -L\left(\kappa \right)}\sin^{2}\theta \right),$$

where $\eta_{0}$ is the viscosity of the carrier liquid and θ denotes the angle between the magnetic field and the vorticity of the flow. The second term in the bracket of Equation (6) is the classical result of the Einstein theory of the effective viscosity of dilute suspensions of hard spheres; the third term corresponds to the so-called rotational viscosity, which appears due to the hindrance of particle rotation in the suspension shear flow by an applied magnetic field [61,62,63,64,65]. Note that Equation (6) was obtained under the assumption that the characteristic time of the particle’s Neel remagnetization is much larger than the time required for the Brownian relaxation of the particles, and the magnetic moment is rigidly linked with the particle body. For typical ferrofluids with iron-oxide particles, this condition is fulfilled if the particle diameter is more than 15–20 nm. This is a typical size for the particles that experience magnetism-induced structuring in the ferrofluids. Equation (6) is good at describing experiments with dilute suspensions of the rigid dipoles (see, for example, [66]), and is often used for the interpretation of experiments with ferrofluids.

Equation (6) predicts the field-induced increase of the relative viscosity:

$$\left[\eta \right]=\left[\eta \left(H\right)-\eta \left(0\right)\right]/\eta \left(0\right),$$

where η(H) denotes the fluid viscosity under the effect of field *H* and $\eta \left(0\right)$ denotes viscosity without a field.

**Figure 4:**
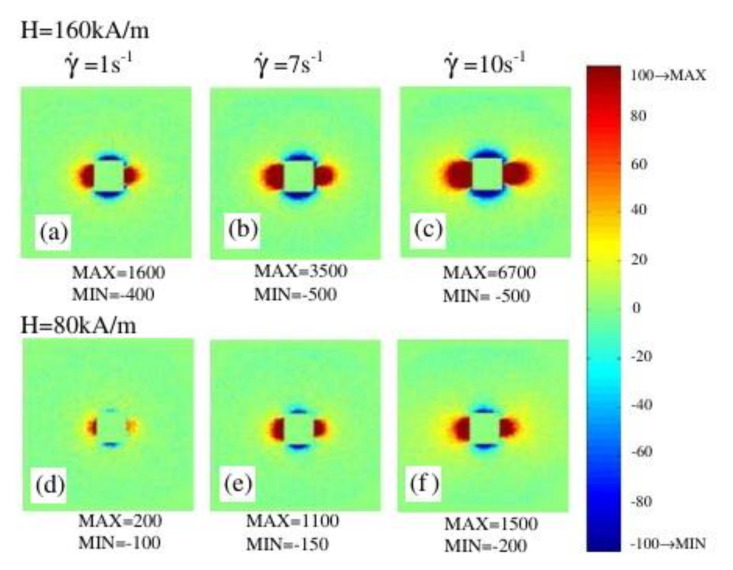
(**a**–**f**) Patterns obtained using small-angle neutron scattering for a ferrofluid based on cobalt nanoparticles for different magnetic field strengths and shear rates. The image was sourced from [77], copyright Elsevier.

**Figure 5:**
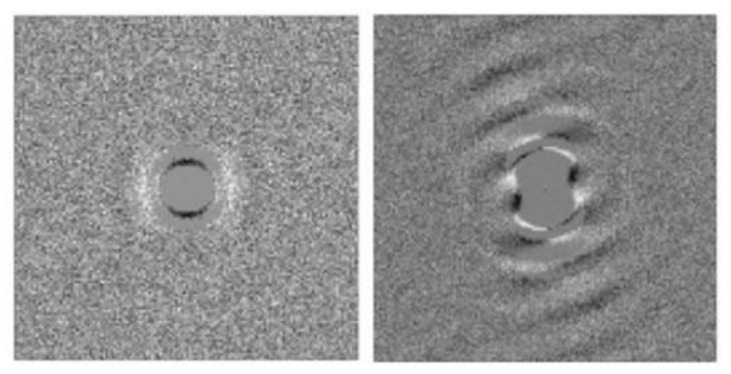
Scattering patterns calculated using Monte Carlo simulations for an idealized monodisperse ferrofluid at rest and experiencing a shear rate of 1 s^−1^ with a magnetic field strength of 200 mT applied for two different directions between a neutron beam and a magnetic field. The images were kindly provided by Patrick Ilg (University of Reading) and were obtained using the methods given in [66].

**Figure 6:**
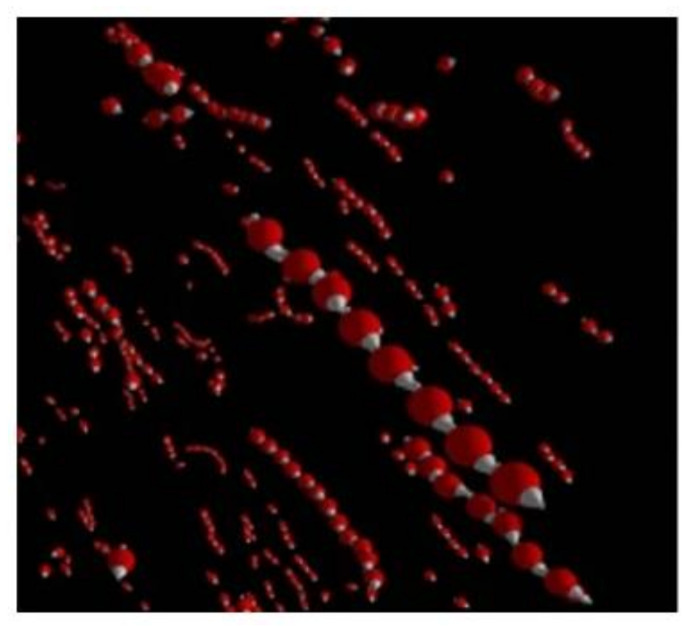
Particle structures obtained from Monte Carlo simulations that were used for calculating the pattern given in Figure 4. The mage was kindly provided by Patrick Ilg (University of Reading) and was obtained using the methods given in [66].

For the typical values of the concentration φ in the order of several percent, the maximal relative increase ${\left[\eta \right]}_{max}\;=1.5\varphi /\left(1+2.5\varphi \right)$ is also about several percent. For typical ferrofluids, this saturation is expected at field strengths of about 200–500 kA/m (see, for example, Figure 1 in [66]). However, the experiments presented in [67,68,69,70,71,72] demonstrate that the field-induced viscosity $\left[\eta \right]$ can achieve an increase of two orders of magnitudes at the field about 100 kA/m. This is three to four orders of magnitude more than the predictions of models [40] of the single non-interacting particles. This shows that the effects of the interaction between the particles and their structuring can play a decisive role in the formation of the macroscopic properties of typical ferrofluids. Experimentally, the formation of anisotropic structures in ferrofluids placed to a magnetic field was observed using small-angle neutron scattering [73,74,75,76,77,78]. Figure 4 shows the experimentally obtained [77] scattering patterns for a ferrofluid based on cobalt nanoparticles experiencing different magnetic field strengths and shear rates. The observed anisotropy of the scattering patterns was excellently reproduced in the Monte Carlo simulations performed in [66]. Figure 5 shows the result of these simulations, while Figure 6 shows the structural image obtained from the simulations. Thus, by combining the scattering experiments and Monte Carlo simulations, it was confirmed that the chain formation of magnetic particles is the reason for the magnetoviscous effects.

In this part of the review, we discuss the works on the chain-like heterogeneous aggregates on the rheological and magnetic properties of these systems.

### 3.1. Magnetoviscous Effect

The physical explanation of the strong magnetorheological effects in ferrofluids is given in [67]. The concept behind this explanation is that real ferrofluids are polydisperse systems, which very often have a wide distribution of particle sizes (corresponding diagrams for various ferrofluids can be found, for example, in [67,68,69,70,71,72,79]). As noted in the Introduction, the main part of the particles in typical ferrofluids is usually too small and unable to aggregate. However, the larger particles in the distribution can be large enough to form heterogeneous clusters and structures under the force of magnetic interaction.

**Figure 7:**
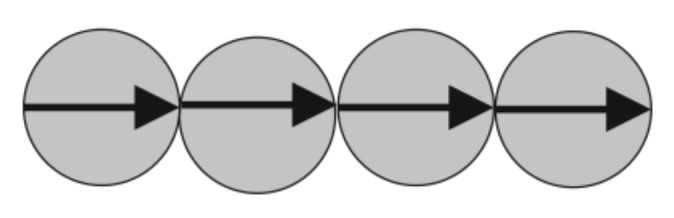
Illustration of a linear chain. The arrows indicate the magnetic moments of the particles.

The simplest kind of the heterogeneous structures in ferrofluids are linear chains, where the particles are aligned “head to tails.” An example of such a chain is illustrated in Figure 7.

A macroscopic shear flow destroys the chain. Analysis has shown that the longer the chain, the lower the force required for destruction. The maximum number of particles in the chain, as determined by the balance between the magnetic attractive force and the destructing force, has been estimated in [67] as:

(7) $$n_{max}=\sqrt{\frac{\mu_{0}}{18\eta_{0}\dot{\gamma }}}M_{p}{\left(\frac{d_{p}}{d_{p}+2s\;}\right)}^{3},$$

where $\dot{\gamma }$ is the shear rate of the ferrofluid flow, $M_{p}$ is the magnetization of the particle material, and *s* is thickness of the stabilizing layer on the particle.

A theoretical model of the effect of the magnetoviscous effect on the chains in ferrofluids was suggested in [68]. For a maximal simplification of the calculations, the bidisperse model of the fluid was used. In this model, the ferrofluid was considered to contain particles of two sizes. The “small” particles that had a diameter that was close to the mean diameter of the particles in the system were considered to be unable to aggregate. The chains were assumed to consist of big particles in the model, whose size and concentration were fit by comparing theoretical and experimental results. The simplest approximation of the chains as straight rod-like aggregates (see Figure 7) was used in this model. This means that any fluctuations of the particles’ positions and orientations inside the chain were ignored. The particles’ magnetic moments were aligned along the chain axis. This means that the energy of the interactions between the particles in the chain was assumed to be much larger than the Zeeman energy of the interaction of each particle’s magnetic moment with an external magnetic field. Diagrams of the size distribution of the particles in the studied ferrofluids demonstrate that the volume concentration of the particles with the large diameters was very small. This is why any interactions between the chains are ignored.

Estimates show that the Peclet number, which is calculated using the realistic values of the shear rate $\dot{\gamma }$ and the size of the chains, is much less than one. Therefore, in the first approximation, the systems of chains could be considered using methods of equilibrium statistical thermodynamics of heterogeneous fluctuations. To this end, the free energy *F* of the unit volume of an ensemble of the particles is presented in the form:

(8) $$F\left[g_{n}\right]=kT\overset{n_{max}}{\underset{n=1}{\sum }}\left[g_{n}\ln\frac{g_{n}v_{l}}{e}+g_{n}f_{n}\right],$$

where $g_{n}$ is the number of *n*-particle chains in the unit volume, $v_{l}$ stands for the volume of the large particle, *e* is Euler’s number (≈2.72), and $f_{n}$ represents the chain’s dimensionless “internal” free energy. In the quasi-equilibrium approximation, the distribution function $g_{n}$ corresponds to the minimum of the functional $F\left[g_{n}\right]$ under the condition of conserving particles:

(9) $$\overset{n_{max}}{\underset{n=1}{\sum }}ng_{n}=\frac{\varphi_{l}}{v_{l}},$$

where $\varphi_{l}$ is the volume concentration of the large particles, and $\varphi_{l}$ and $v_{l}$ are to be estimated from the comparison of the theoretical and experimental results. By using the standard procedure, one obtains:

(10) $$g_{n}=\frac{X^{n}}{v_{l}}\exp\left(-f_{n}\right),$$

where *X* is the Lagrange undetermined parameter, which can be found by substituting Equation (10) into Equations (9). For the chains as straight rods, the internal free energy of the chain was estimated as [68]:

(11) $$f_{n}=-\varepsilon_{l}\left(n-1\right)-ln\left(\frac{\sinh\left(\kappa_{l}n\right)}{\kappa_{l}n}\right),\varepsilon_{l}=\frac{\mu_{0}}{2\pi }\frac{M_{p}^{2}v_{l}^{2}}{{\left(d_{l}+2s\right)}^{3}kT},\kappa_{l}=\mu_{0}\frac{M_{p}v_{l}H}{kT}.$$

Based on the approximations used, the ferrofluid can be considered as a suspension of the magnetic rods with the distribution function $g_{n}$ over their length. The macroscopic stress σ in the suspension consists of two parts: the symmetrical part $\sigma^{s}$ and the antisymmetric part $\sigma^{a}$ relative to the components of the tensor of the gradient of the velocity flow. The $\sigma^{s}$ part represents the stress in the suspension of the rod-like structures and appears because of perturbations induced by the rods in the suspension flow. The antisymmetric part $\sigma^{a}$ is induced by magnetic torques that act on the chains such that they are deviated by the flow away from the field direction. Both parts of the stress σ are found using the function $g_{n}$ and the angle of deviation of the chain from an applied magnetic field. Due to Brownian effects, the distribution function of the deviation angle must be found using the solution of the corresponding Fokker–Plank equation. The details of the solution of this equation and the determination of the stress σ are given in [68]. Once the stress is found, one can find the suspension’s effective viscosity $\eta =\sigma /\dot{\gamma }$ in terms of the field *H*, shear rate $\dot{\gamma }$, and the volume $v_{l}$ and concentration $\varphi_{l}$ of the large particles.

**Figure 8:**
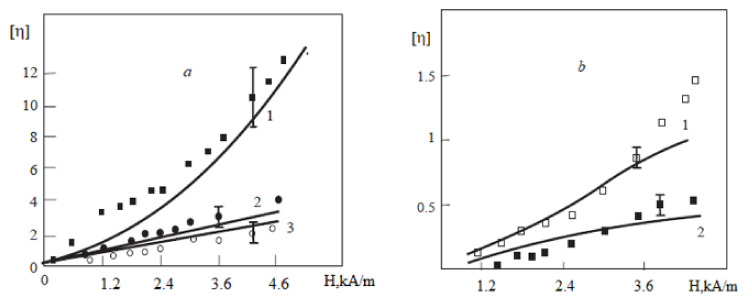
The dependence of the reduced magnetic effective viscosity on magnetic field *H* inside the ferrofluid [68]. The diameter of the magnetic core of the ‘‘large’’ particles was $d_{l}=16.5$ nm, while their hydrodynamic volume concentration was $\varphi_{l}=0.017$. The dots are experimental data, while the lines correspond to calculations: (**a**) $\dot{\gamma }=0.1\;s^{-1}$ (1), $0.5{\;s}^{-1}$ (2), and $0.9\;s^{-1}$ (3); (**b**) $\dot{\gamma }=1.05\;s^{-1}$ (1) and $5.23{\;s}^{-1}$ (2).

Some results of the calculations and measurements [68] of the relative viscosity $\left[\eta \right]$ are presented in Figure 8. The diameter $d_{l}$ and volume concentration $\varphi_{l}$ of the large particles that the chains consist of were estimated using the best agreement of the theoretical and experimental results at the minimal shear rate of $\dot{\gamma }=0.1{\;s}^{-1}.$ Then, these values of $d_{l}$ and $\varphi_{l}$ were used for the calculations at the other shear rates. The model [68] has also been used for describing the experimental data found in [70,72], and a good agreement between theory and experiments was achieved.

It should be noted that the approach used in [68] was developed under the assumption that the energy of the magnetic attraction between the nearest particles in the chain was significantly larger than the Zeeman energy of the interaction between the particle magnetic moment and the applied field. This means that the inequality $\varepsilon_{l}>\kappa_{l}$ was assumed. Estimates show that for a particle diameter $d_{l}$ of about 20 nm and a surfactant layer thickness *s ≈* 2 nm, the inequality holds for a magnetic field strength that is less than 20–25 kA/m. Experiments with significantly stronger magnetic fields are reported in [66,80,81]. The strong magnetoviscous effect, as well as the fast decrease in the viscosity with the shear, was detected in these studies. In [66], one can find a comparison between the theoretical, computational, and experimental results for the magnetoviscous effect in the cobalt ferrofluids in a range of magnetic field strengths up to 130 kA/m and at high shear rates that were able to destroy the chains.

The idea that the fractions of the biggest particles of ferrofluids play a decisive role in the rheological properties of these systems has been checked in experiments [82,83,84]. In these works, the fractions of the relatively big and relatively small particles were separated using the magnetic gradient method [83] and centrifugation [84]. The magnetoviscous effects in the original fluids and in the fluids with high and low concentrations of the big particles differed by up to an order of magnitude.

The magnetoviscous effect in magnetite and cobalt-based ferrofluids was compared using experiments [85]. The magnetite ferrofluid contained mainly small particles with weak magnetic interparticle interactions. The probability of structure formation in this fluid was low and the measured relative change of viscosity did not exceed 0.5. In contrast, the cobalt-based ferrofluid with strongly interacting particles demonstrated a significant magnetoviscous effect. This agrees with the discussed chain formation model [68], where the appearance of the magnetoviscous effect is attributed to the strong interparticle interaction in the presence of a magnetic field. Strong anisotropy of the magnetoviscous effect at various orientations of the applied magnetic field was detected in experiments [86] and computer simulations [87]. This anisotropy can only be explained by the formation of the internal heterogeneous structures under the action of a field. The true nature of these structures, i.e., whether there were chains or bulk aggregates, was not established.

Computer simulations of chain structures in ferrofluids involved in macroscopic shear flow were performed in [88,89]. The function $g_{n}$ of the chains’ size distribution for various magnetic fields, the chains average length, and their effect on macroscopic properties of the fluid were evaluated. The magnetoviscous effect was much stronger than that found using the theory [61] modeling single particles; the reduction of the magnetoviscous effect with the macroscopic shear rate $\dot{\gamma }$ was also found in the simulations. The obtained results can serve as a basis for a more detailed theoretical analyses of microscopic mechanisms of the rheology of field-structured magnetic colloids.

### 3.2. Viscoelastic Effects

If the shear rate $\dot{\gamma }$ of a ferrofluid changes, the internal structures do not adopt their new values instantly, but instead after some time. This leads to retardation of the stress σ alteration, i.e., to the viscoelastic effects. Experiments [70] have demonstrated that in typical magnetite ferrofluids, the characteristic time τ of the viscoelastic relaxation increases with the magnetic field, and it varies in the range of several seconds for field strengths in the range 4–14 kA/m. It is possible that the first attempt to describe the viscoelastic phenomena base on a microscopic model of a ferrofluid was made in [90]. Similarly to [68], the system of rod-like chains of the magnetic particles was considered in this work. The supposed relaxation mechanism was the finite rate of kinetics of the chain’s reorientation in the alternating shear flow. Unlike the experiments [70], the model [90] predicts the decrease of the relaxation time τ with a strengthening applied magnetic field; the estimate of $\tau \;\sim \;10^{-4}\;s$ follows from the results of [90]. This means that the supposed relaxation mechanism of the chain’s reorientation is not accurate for the physical situation in the ferrofluids under study.

Results of the viscosity prediction made in [90] were compared with results obtained by varying the dipolar interaction strength using the Langevin dynamics simulations in [91]. Qualitatively, the results of the theoretical model and simulation were consistent, but at the same time, with large interaction forces, there was an obvious quantitative difference.

**Figure 9:**
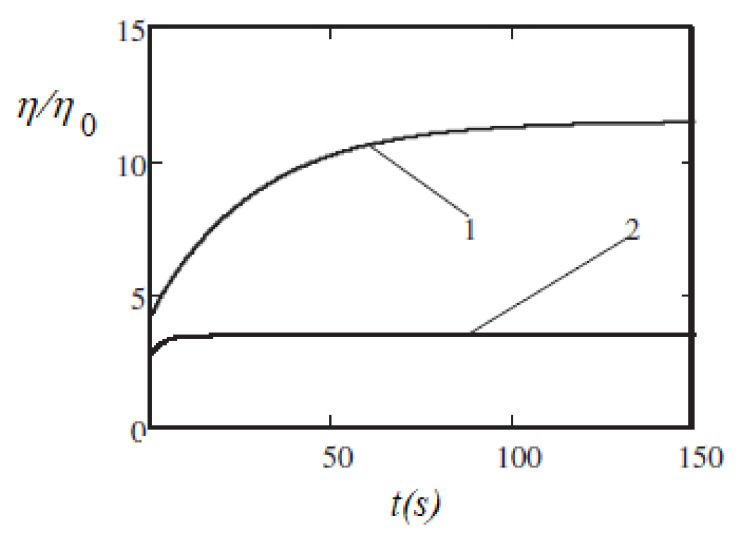
The time dependence [92] of the effective viscosity η after a stepwise decrease of the shear rate $\dot{\gamma }$ from 16 s^−1^ to 1.6 s^−1^ at *t* = 0. Curve 1—the dimensionless magnetic field κ=3 Curve 2—κ=1. Parameters of the system: the hydrodynamic diameter (with the surface layers) of the particles was 16 nm, the volume concentration of the particles was φ=  0.015, the dipolar coupling was λ  = 2.75, the viscosity of the carrier liquid $\eta_{0}=0.13$ Pa∙s. Published with the permission of the American Physical Society (license RNP/20/AUG/029407).

Another model of the viscoelasticity was suggested in [92]. This model is based on the concept that the retardation effects take place because of the finite rate of kinetics of the chain’s formation–destruction. Figure 9 illustrates the results of calculations of the evolution of a ferrofluid’s viscosity η after a stepwise change of the shear rate $\dot{\gamma }$.

**Figure 10:**
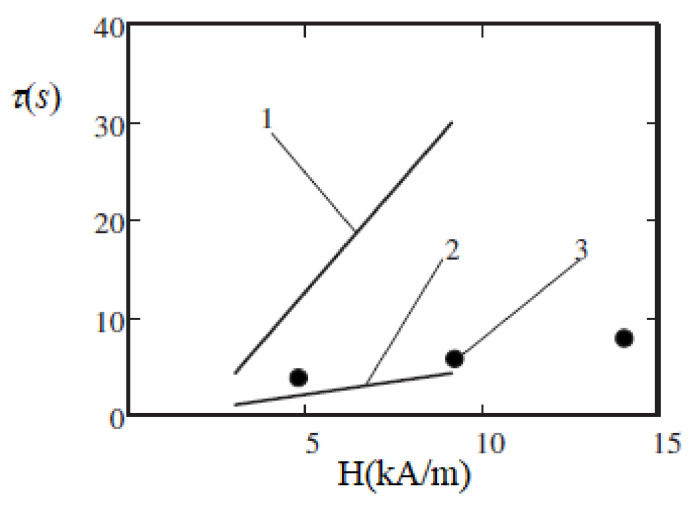
Relaxation time τ versus the applied magnetic field *H*. Curves 1 and 2—theoretical calculations [92] for a ferrofluid with the same parameters as in Figure 9. Theory: the shear rate $\dot{\gamma }$ changed in a stepwise manner from 16 s^−1^ to 1.6 s^−1^ (line 1) and back (line 2). Dots 3—experiments [70] with an oscillating shear flow. Published with the permission of the American Physical Society (license RNP/20/AUG/029408).

The results show that when the applied field increased from κ=1 to κ=3, the characteristic time τ of the viscosity relaxation varied from several seconds to several tens of seconds. This is, at least qualitatively and in the order of magnitude, in agreement with the experiments [70]. Some results of the time τ calculations [92] and its measurements in [70] are shown in Figure 10. In the experiments [70], the relaxation time τ was determined using the relation $\tau =2\pi /\omega_{max}$, where $\omega_{max}$ is the frequency of the shear rate oscillations, which corresponds to the maximum of the imaginary part of the complex viscosity. In [92], a stepwise change of the shear rate $\dot{\gamma }$ was supposed. The measured results [70] for τ were between the theoretical results for the instantaneous increase and decrease of $\dot{\gamma }$. This shows that the model [92] was adequate, at least, in terms of its main physical points. Of course, the polydispersity of the real ferrofluid and the monodispersity of the model [92], as well as the simplification of the chains as straight rods, influenced the quantitative agreement between the theoretical and experimental results. Note that the relaxation time τ, which corresponds to the decrease of $\dot{\gamma }$ (curve 1 in Figure 10), was significantly larger than that corresponding to the increase of $\dot{\gamma }$ (curve 2). This was expected since a decrease in the shear rate leads to the growth of the chains, whereas an increase in the shear rate leads to their rupture. Of course, the former process requires a much longer time than the latter one. Additionally, some other experimental works have addressed the issue of the relaxation of rheological properties in ferrofluids (see, e.g., [93,94,95]), but systematic studies are required to significantly improve the theoretical modeling.

## 4. Condensation Phase Transitions in Ferrofluids

We have noted that the particles of ferrofluids can experience a phase condensation into bulk dense phases [20,30,31,32,33,34,36,38,39,40,41,42,43,44,45,46,47,48,49,50,51,52,53,54,55] that is similar to the first-order phase transitions in molecular and other colloidal systems. This effect is accompanied by the formation of highly concentrated, drop-like aggregates that are suspended in a surrounding liquid and diluted with colloidal ferroparticles. The droplets are enriched with ferroparticles, where the volume fraction of which may reach 50–60%. An applied magnetic field stimulates this condensation and elongates the new phase nuclei along its force lines. Due to the very weak interfacial tension (~10^−6^ N/m [39,40,41,43,52,54]), the elongation degree might be very large, even under the influence of a weak-to-moderate strength external field; therefore, the experimental observations will very often demonstrate the columnar-like structures, as shown, for example, in Figure 1.

The first theoretical models [57,58,96,97] of this phenomenon treated it as “gas–liquid” phase transition in the system of the magnetic particles, similar to the classical van der Waals transition in systems with a central interparticle interaction. However, direct TEM observations [16,17,98] have shown that the linear chains, branched forks, closed rings, nets, and other structures that consist of the chain segments can appear in these systems at the stage preceding the phase separation.

**Figure 11:**
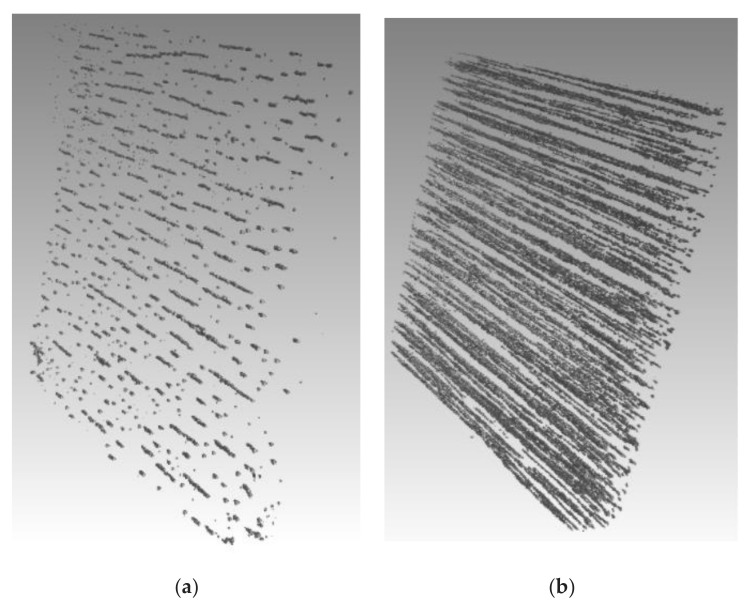
Three-dimensional visualization of the (**a**) short chains (obtained at *H* = 10 kA/m) and (**b**) bulk structures (obtained at *H* = 450 kA/m) of magnetic particles. The images were kindly provided by Dmitry Borin (TU Dresden) and were obtained using methods equivalent to those given in [103].

**Figure 12:**
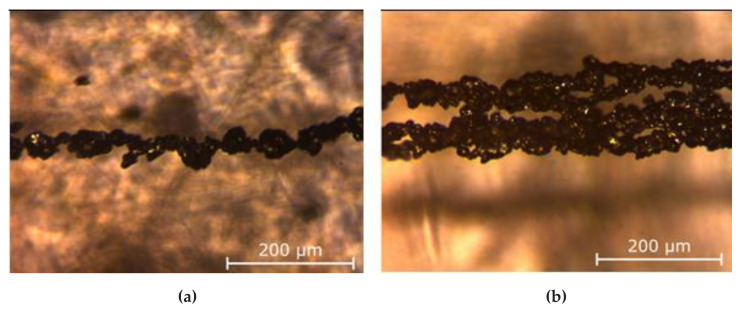
Microscopic images of a single thin chain (**a**) and bulk structure/thick chain (**b**) of magnetic particles similar to the structures shown in Figure 11. The images were kindly provided by Dmitry Borin (TU Dresden) and were obtained using methods equivalent to those given in [103] and shown in Figure 11. Adopted from [103].

The fundamental mechanisms of the phase transitions in ferrofluid are still not well understood. The principal question concerns whether the dipole forces, without a fully screened central colloidal interparticle attraction, can induce the bulk phase condensation of the particles. There seems to be no doubt that the bulk condensation in the systems of purely dipole–dipole interacting particles can take place under the action of a strong enough magnetic field with the magnetic moments of all particles being well oriented. Indeed, the field-induced bulk structures have been detected not only in laboratory experiments [17,20,30,31,32,33,34,36,38,39,40,41,42,43,44,45,46,47,48,49,50,51,52,53,54,55], where the details of the interparticle interaction were not completely known, but also in computer simulations [18,99,100,101] with dipole–dipole interacting particles. Moreover, these transitions have been directly observed many times in experiments with magnetorheological suspensions that consist of micron-sized magnetizable particles (for an overview, see [102]). Figure 11 and Figure 12 visualize a real microstructure of magnetic microparticles assembled as short single chains and as bulk structures, as reported in [103].

The most disputable point is the problem of the phase condensation of the dipole particles without the applied field. The conclusion that, at *H* = 0, the increase of the dipolar coupling parameter λ and the particles’ concentration φ leads to the appearance of chains first and then to topologically more complicated branched and net-like structures was reached in theoretical works [104,105,106] and computer simulations [19,20,107,108]. At the same time, the gas–liquid phase transitions in Stockmayer fluids, i.e., fluids with the particles interacting both with the dipole–dipole and through the central attraction, was observed in many computer simulations [109,110,111,112,113,114].

Another point of view is reported in theoretical models [115,116], in which the main focus was put on the formation of linear rod-like chains in low-concentration ferrofluids at high values of λ. Analysis of these works shows that, because of the magnetic interaction between the chains, their mean length was non-monotonic with φ, i.e., they had a maximum. Note that a chain length reduction, starting with some certain concentration of the particles, was detected in the computer simulations [117]. According to [115,116], an increase of either φ or λ results in chain shortening, which induces their condensation into dense phases. From this point of view, the absence of the bulk phase separations in the systems with the pure dipole–dipole interparticle interactions can be explained by the hypothesis that, in the conditions of the computer simulations, the appearance of the supercritical nuclei of the dense phase took much longer than the simulation time. A strong enough central attraction between the particles can significantly reduce this time, where the phase condensation was observed in Stockmayer fluids due to this reduction.

In computer simulations [118], quasi-spherical dense nuclei consisting of particles with a pure dipole–dipole interaction were created artificially at the onset of the simulation. These nuclei were surrounded by a “vapor” of the particles and there was no magnetic field. The simulations demonstrated the tendency of the dense globules to grow, which can be considered evidence for the physical possibility of particles’ phase condensation without an applied field. Nevertheless, the question regarding the fundamental mechanisms of the phase transition is still open and worth investigating further.

The kinetics of the bulk phase separations in ferrofluids has been studied poorly. The earlier works (see [56,119] and references therein) interpret this process as a homogeneous condensation of a “gas” of identical magnetic particles in “liquid” drops. The existence of the chains and other heterogeneous aggregates, as well as the possibility of the heterogeneous scenario of nucleation on the biggest particles of ferrofluid, were not considered in this approach. It was supposed that at the latest stage of the condensation, the “drops” coalesced according to the classical Ostwald ripening scenario. Experiments [32] have demonstrated that the drop evolution in an applied magnetic field can include at least two stages. In the first stage, the drops grow due to the usual mechanism of adsorption of the single particles from the surrounding “gas.” In the second stage, the drops coalescence because of their magnetic attraction. A theoretical model of the two-stage evolution has been suggested in [32], where the results were in agreement with the experiments.

### 4.1. Effect of the Drop Aggregates on the Viscous Properties of Ferrofluids

In the motionless ferrofluids, the “drops” of a new phase coalesce because of either the Ostwald ripening or the magnetic attraction scenario and can grow up to percolate through the container with the system. Note that the drop aspect ratio (the ratio of the major to the minor axis) increases with the drop volume [40,56]. If the phase-separated ferrofluid under the action of an external field is involved in a macroscopic shear flow, the hydrodynamic forces rupture the most elongated drops with the largest volume. Therefore, the shear flow restricts the drop growth to some certain volume and the ferrofluid presents an emulsion of the drops with a given size and aspect ratio. Strong magnetoviscous effects in ferrofluids with the bulk aggregates have been detected in experiments using TEM methods [81]. A theoretical description of these results has not been achieved yet.

The main problem facing the microscopic analysis of the rheological effects in ferrofluids with drop-like aggregates is to understand the mechanisms of the drop’s destruction due to the hydrodynamic forces. Various models of the similar rupture effect with different mechanisms of the drops’ destruction have been suggested for magnetorheological suspensions of micron-sized magnetizable particles [120,121,122,123]. In these models, the total viscous stress σ in the suspension is presented as:

(12) $$\sigma =\eta_{0}\dot{\gamma }\left(1+C\Phi Ma^{-p}\right),$$

where *Φ* is volume concentration of the aggregates and $Ma=\eta_{0}\dot{\gamma }/\mu_{0}H^{2}$ is the so-called Mason number, which is proportional to the ratio of the hydrodynamic forces to the magnetic forces acting on the drop-like aggregates. The second term in the brackets represents the effect of the drops on the suspension viscosity. The magnitudes of the multiplier *C* and exponent *p* were determined differently in [120,121,122,123]: the model in [120] predicts Bingham-like behavior of the suspension with p=1, the models in [121,122] give p=2/3, whereas the approach in [123] predicts p=4/5. A comparison of these models with experiments [123,124] does not allow us to choose the best of these models. Thus, the internal mechanism of formation of the macroscopic magnetoviscous effect in magnetorheological suspensions with the bulk structures remains undiscovered. In ferrofluids, the theoretical problem is more complicated because of the intensive Brownian motion of the particles.

A small-angle light-scattering method of observation of dependence of the size of the internal structures in ferrofluids on time, magnetic field and shear rate has been developed in [125]. This method opens broad perspectives that can be used to obtain microstructural information, to evaluate structuring kinetics, and to determine the relationships between these internal transformations and the magnetic and rheological properties of the fluids. In experiments [126], ferrofluids with oxide iron nanoparticles were diluted by water and sheep’s blood. This work aimed to develop a scientific background for biomedical applications of ferrofluids, in particular, for cancer therapy. Note that the dense bulk aggregates that were observed in these fluids were subjected to an external magnetic field. The experiments showed that the magnetoviscous effect in the ferrofluid diluted by the blood was significantly stronger than that in the same fluid diluted by water in the same proportions. This fact demonstrates the interaction of the formed structures with the red blood cells, resulting in the enhancement of the magnetoviscous effect. This must be taken into account in potential biomedical applications of ferrofluids.

**Figure 13:**
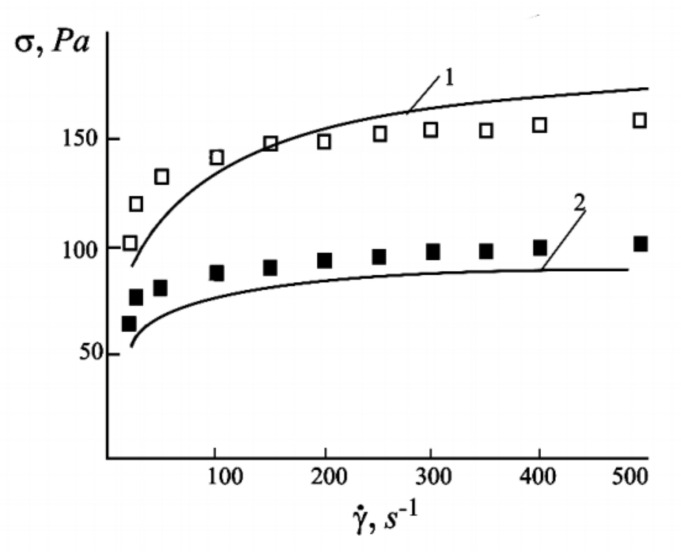
Total stress *σ* versus the shear rate $\dot{\gamma }$. Lines represent theoretical predictions and the dots represent experimental data: line 1 and open squares correspond to a field of 8.6 kA/m, while line 2 and filled squares correspond to a field of 5.7 kA/m. Adopted from [122]. Further details are given in [122].

The models used in [120,121,122,123], as well as the interpretations of the experiments in [125,126] were based on the idea that the macroscopic rheological properties of the ferrofluids are determined by the internal dense spindle-like aggregates. Figure 13 demonstrates a high correspondence between the experimentally measured and theoretically predicted total shear stress *σ* in a ferrofluid under shear flow and the shear rate $\dot{\gamma }$ at two different magnetic field strengths.

**Figure 14:**
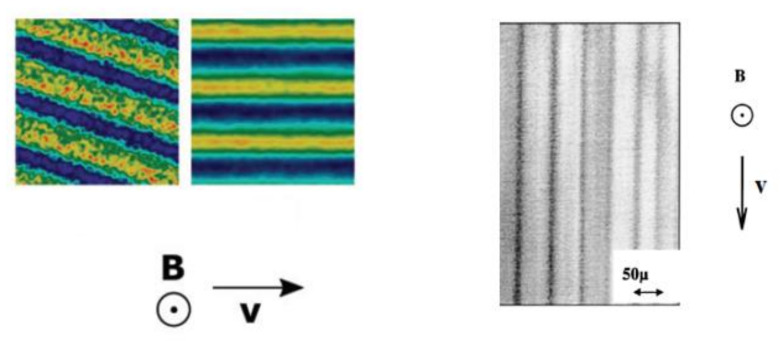
Lamellar structures detected in computer simulations [88] (left; Figure 17 of [88]) and in laboratory experiments [127] (right, Figure 3 of [127]). Published with the permission of the Royal Society of Chemistry (license 1055535-1) and American Physical Society (license RNP/20/AUG/029406).

However, computer simulations [88] have demonstrated that in a shear flow condition, the discrete aggregates can transform into lamellar structures parallel to the flow. A similar structures has been detected in magnetorheological suspensions [127] (see Figure 14). This effect must be taken into account during the theoretical modeling and interpretation of experimental results.

### 4.2. Quasi-Elastic and Yield Stress Effects

When the bulk aggregates percolate the container with a magnetic fluid and form a bridge between the opposite boundaries of the container [59], the systems can experience static elastic deformation under the imposed shear stress. If the stress exceeds some threshold value, called the yield stress, the structures rupture and the mechanical reaction of the fluid changes from the elastic regime to the flow regime. These phenomena are strong and were very well measured in the magnetorheological suspensions (see, for example, an overview in [102]). In a magnetic field with a strength of about 100 kA/m, the yield stress in some magnetorheological suspension samples can achieve several tens of kilopascals [128]. The yield stress in ferrofluids with the nanoparticles can also be significant. As an example, the stress in ferrofluids with a 5% volume fraction of Co particles achieved several hundred pascals [129]. The effect of particle shape on the yield in ferrofluids was studied in [130]. The measured stress in the system with the fiber particles was an order of magnitude larger than that in the systems with the spherical particles.

A theoretical model of the yield stress in ferrofluids, based on the idea of the rupture of the bridging structures, was suggested in [131]. Two microscopic mechanisms of transition from the elastic to fluid behavior of the ferrofluid were analyzed. The first is related to the loss of the mechanical equilibrium of the domains, which are sloped under the shear stress relative to the direction of the externally applied magnetic field. The second mechanism is connected to the breakup of the “bridge” into two separate drops when the shear strain exceeds a critical magnitude. Estimates show that for real ferrofluids, the second mechanism is more probable.

In particular, it was shown that the yield stress $\sigma_{y}$ must decrease with thickness *L* of the gap filled by the fluid, according to the scaling relation $\sigma_{y}\sim L^{-1/3}$. The results agree with the measurements [132,133,134] of the stress $\sigma_{y}$ in magnetite ferrofluids. One needs to note that the results of [134] demonstrate a low yield stress $\sigma_{y}$, which at *H* = 20 kA/m, was in the order of magnitude of 0.1 Pa. The yield stress for a cobalt-based ferrofluid placed in the field with the same strength can be significantly higher, reaching several pascals [85,135].

## 5. Ferrofluids with Multicore Particles

**Figure 15:**
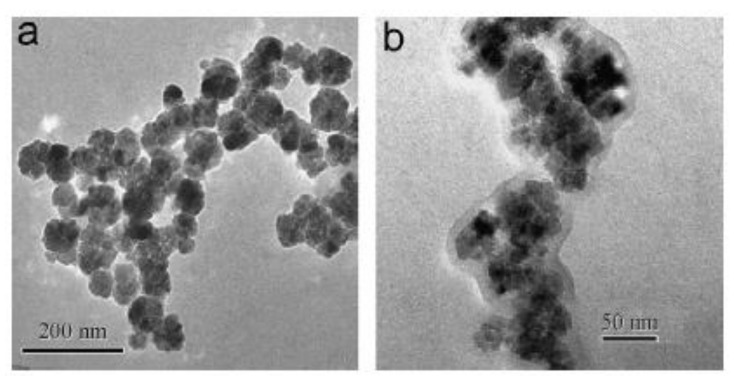
TEM images [137] of the clustered particles for two different samples. The mean diameter of the magnetic nanoparticles is 13 nm (**a**) and 17 nm (**b**).

Within the last decade, interest in ferrofluids with multicore particles [136] has been growing due to their potential applications in high-tech and biomedical applications, such as the magnetic hyperthermia of oncological diseases [137,138,139] and various damper systems [140]. Clustered multicore magnetic particles consist of single-domain particles with a typical diameter of about 10 nm that are “clued” by a polymer or other nonmagnetic shells. The typical size of the multicore particles is about 100 nm. Some photos of the particles, synthesized in [136], are shown in Figure 15. Other examples of multicore particles with almost regular spherical shapes are presented in [141,142]. The potential efficiency of the particles for the magnetic hyperthermia method of cancer therapy has been demonstrated in [139].

**Figure 16:**
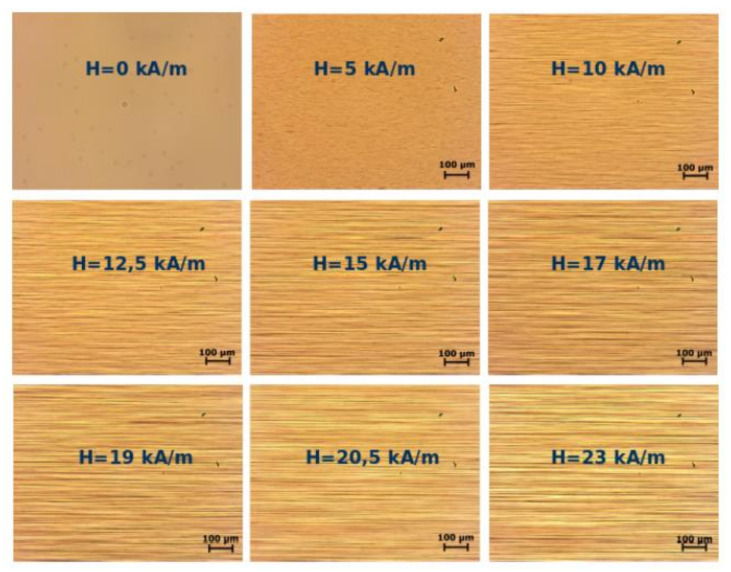
Microscopic images of the magnetic fluid based on multicore magnetite nanoparticle structures at various magnetic field strengths; the concentration of the magnetic phase was *φ ≈* 0.013 vol.% [125].

The typical size of multicore particles is between the size of particles in standard ferrofluids and in magnetorheological suspensions. Therefore, their physical properties occupy an intermediate position between the properties of these two systems. In part, the multicore-based ferrofluids are more stable than the standard magnetorheological suspension with the micron-sized particles. At the same time, they demonstrate magnetorheological effects that are much stronger than those in the standard ferrofluids with single-core nano-sized particles. The combination of the stability with the high response to an applied field appears to be very promising for many applications. The strong (about two orders of magnitude higher) magnetoviscous effect in these fluids placed in the field ranged of about 30 kA/m was detected in [99]. This effect has been explained by considering the unification of the multicore particles into chain-like aggregates, whose length was restricted by the viscous hydrodynamic forces in the flowing ferrofluid. The size of aggregates formed by multicore particles in an externally applied magnetic field allowed for observing them using conventional optical microscopy (Figure 16).

**Figure 17:**
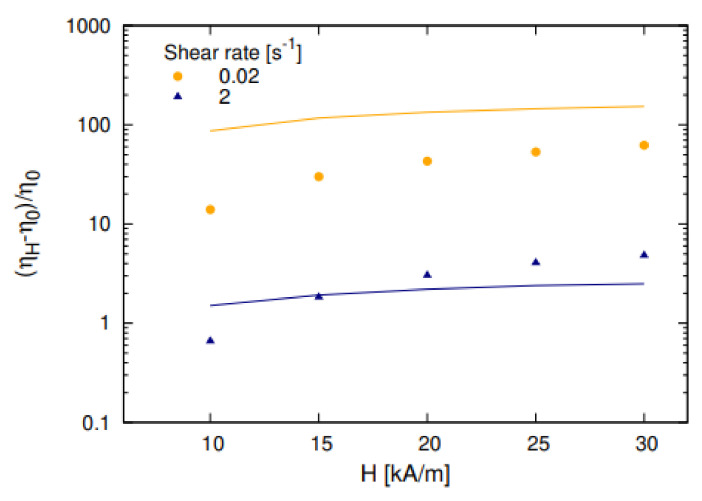
The magnetoviscous effect in a multicore-based ferrofluid was measured and calculated using various shear rates as a function of the applied magnetic field. The solid lines are the results of the calculations performed according to the chain model (hydrodynamic diameter of the particle cluster was assumed to be 90 nm, *λ* = 2.3) [143].

TEM images of the chain formed by multicore microparticles are presented in [141]. Experimental and computer measurements of the rheological properties of the ferrofluids are in good agreement with the chain model. Strong magnetovisocus effect in the fluids with the clustered particles has been detected and theoretically explained in ref. [143]. Some results of this work are presented in Figure 17. 

**Figure 18:**
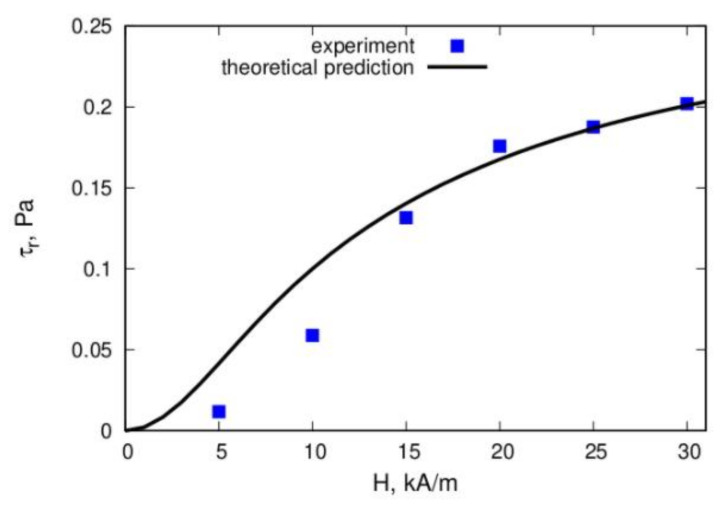
Comparison of the theoretical prediction and experimental results for the residual stress τ_r_ versus the magnetic field strength. The proportion of magnetic material that was part of the bulk drops was 0.12. Details of the experiment and calculations are given in [144]. The plots were redrawn by the authors using our own raw data.

**Figure 19:**
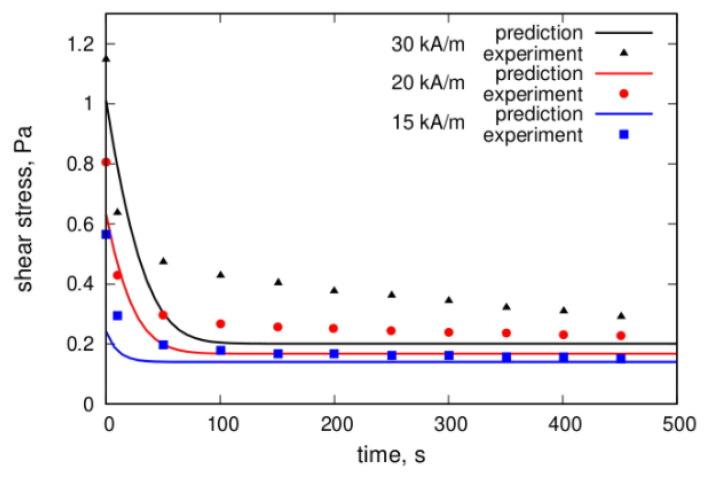
Comparison of the theoretical prediction and experimental results of the stress relaxation for various magnetic field strengths after a stepwise change of the shear rate from 0.02 to 0 s^−1^. Details of the experiment and calculations are given in [144]. The plots were redrawn by the authors using our own raw data.

**Figure 20:**
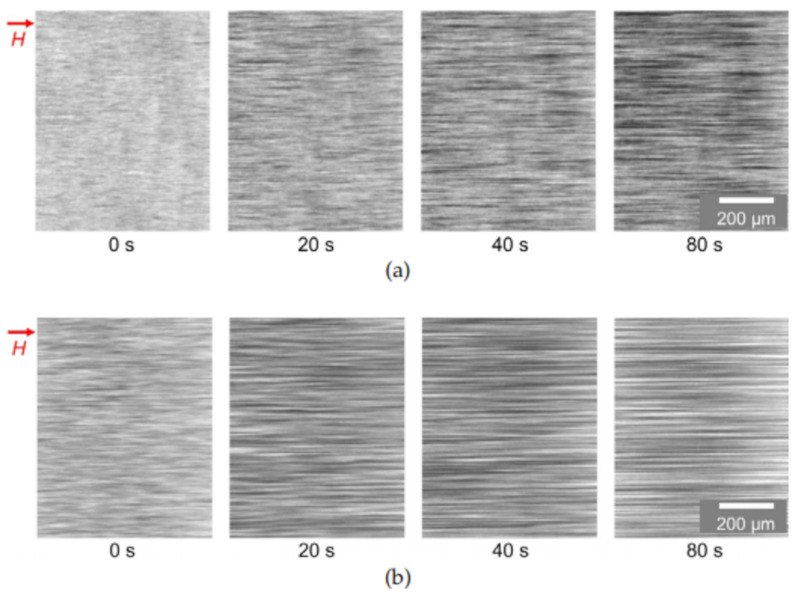
Structures formed by a multicore-based ferrofluid under the influence of a magnetic field of H = 20 kA/m (**a**) and H = 80 kA/m (**b**) for several time steps after the application of the respective magnetic field strength [126].

Slow viscoelastic relaxation, static yield stress, and residual stress, which take place after interruption of the fluid flow, have been detected in experiments [144]. A theoretical explanation [144] of these effects was based on the assumption that the multicore particles form not only the linear chains in the applied field but also bulk dense clusters that can percolate through the measuring container with the fluid. A comparison of theoretical calculations with the experiment is shown in Figure 18 and Figure 19. Direct optical observations of the microstructural changes in multicore-based magnetic fluids that were performed in [126] verified the dependence of the formation rate and size of the particle structures on the applied field strength; this is shown in Figure 20. Further results of experimental studies of the magnetoviscous effect and structure formation in the multicore-based ferrofluids can be found in [142,145,146,147,148,149].

## 6. Conclusions

In conclusion, both types of structural transformations in ferrofluids, namely, the chain formation and the bulk phase separation, have the same physical origin, i.e., they take place due to the interparticle magnetic dipole–dipole interaction. Concerning the chain aggregates, this conclusion was expected since the head-to-tail position of uniformly magnetized nanoparticles is energetically advantageous. The chain distribution over their length is determined by a balance between the energy gain and the entropy loss. Since an external magnetic field results in the alignment of particle magnetic moments, it was expected that the chain formation is intensified by a field strengthening. Of course, the larger the particle magnetic moment, the stronger the chain formation tendency. This means that in real commercial, well-stabilized ferrofluids, the chain aggregates should only be formed between the particles from large size fractions.

Regarding the condensation-like phase separation, the question whether magnetic dipole–dipole interactions are capable of stimulating the particle condensation alone is still an open question. However, similar to the Stockmayer fluid, it was shown [150,151,152] that accounting for the van der Waals colloidal attractive forces (in addition to the magnetic dipolar interaction) could result in predicting breaks in the thermodynamic stability of the homogeneous state of ferrocolloids. Furthermore, the particle polydispersity is crucial here because the phase condensation could only be observed under the condition that a considerable number of rather large particles (the size of which exceeded 18–20 nm) exists in the suspension. The effect is that the van der Waals colloid attraction could be poorly screened by the surfactant layers or by the double electric layers in sterically or electrically stabilized ferrocolloids, respectively. The magnetic interaction plays a supporting role here, manifesting itself mainly under the presence of an external magnetic field. Thus, phase separation is observed primarily as a field-induced effect.

Under the field action, both the magnetic nanoparticle chains and the drop-like aggregates present highly elongated objects that greatly exceed the single nanoparticles. What is more important is that the direction and degree of their elongation are controlled by the direction and strength of the external magnetic field. Additionally, the interplay between these controllable needle-like structures and the hydrodynamic forces in flowing ferrofluids results in the viscous and the viscoelastic peculiarities the present review is devoted to.
